# Is Resin Infiltration a Microinvasive Approach to White Lesions of Calcified Tooth Structures?: A Systemic Review

**DOI:** 10.5005/jp-journals-10005-1579

**Published:** 2019

**Authors:** Vidya Manoharan, S Arun Kumar, Selva B Arumugam, Vijay Anand, Santham Krishnamoorthy, John J Methippara

**Affiliations:** 1 Department of Pedodontics and Preventive Dentistry, Royal Dental College, Palakkad, Kerala, India; 2 Department of Public Health Dentistry, Educare Institute of Dental Sciences, Malappuram, Kerala, India; 3 Department of Paediatric and Preventive Dentistry, Indira Gandhi Institute of Dental Sciences, Puducherry, India; 4 Department of Pedodontics and Preventive Dentistry, Tagore Dental College, Chennai, Tamil Nadu, India; 5 Department of Pedodontics and Preventive Dentistry, Sathyabama Dental College, Chennai, Tamil Nadu, India; 6 Department of Pedodontics and Preventive Dentistry, Annoor Dental College, Muvattupuzha, Kerala, India

**Keywords:** Icon, Minimum intervention dentistry, Resin infiltration, Subsurface caries lesion

## Abstract

**Aim:**

The treatment of white lesions should aim at arresting the lesion progression of carious lesions and improving the esthetics by diminishing the opacity of the developmental disturbances of a tooth. The objective of this review was to present the scientific basis, the principles of resin infiltration and to discuss its inherent clinical applications.

**Data sources:**

Data were identified by PubMed searches. Papers published in English between 2010 and 2015 were selected and most up-to-date or relevant references were chosen.

**Conclusion:**

The resin infiltration technique, while promising, needed more clinical evidence for conclusive findings. However, based on available laboratory and clinical studies, it seems convincing that resin infiltration of enamel lesions should aim at arresting the progression of white spot lesions. Combining this microinvasive approach with a substantial caries remineralization program may provide therapeutic benefits and significantly reduce both long term restorative needs and costs, thus complementing the concept of minimum intervention dentistry.

**How to cite this article:**

Manoharan V, Kumar AS, *et al.* Is Resin Infiltration a Micro invasive Approach to White Lesions of Calcified Tooth Structures?: A Systemic Review. Int J Clin Pediatr Dent 2019;12(1):53–58.

## INTRODUCTION

In recent years, a dramatic change has evolved in the management of dental caries from the traditional restorative treatment approach to a more preventive approach, namely non-invasion or minimal invasion.^1^ Enamel carious lesions are characterized by mineral loss in the body of the lesion, resulting in greater visual enamel opacity due to alteration of the refractive index of the affected area.^2,3^ Great attention has been devoted to the noninvasive treatment of enamel carious lesions, which includes remineralization of the lesion with fluoride and casein phosphopeptide-amorphous calcium phosphate, or the use of therapeutic sealants for occlusal lesions. Fluoride and casein phosphopeptide-amorphous calcium phosphate play a key role in the remineralization of superficial white spot lesions. However, this approach is not always successful as it requires good compliance of the patient, with a change in harmful habits, and with many of the patients abandoning the treatment before completion. Sealants have been used therapeutically on non-cavitated enamel caries as an attempt to reduce lesion progression. The pores within the body of enamel caries provide diffusion pathways for acids and dissolved minerals.^4^ Therefore, an alternative approach for superficial sealing might be based on experiments conducted by Robinson et al.^5^ to arrest carious lesions by the infiltration of these pores with resorcinol-formaldehyde resins. This concept has been modified and commercially developed in Germany for the management of smooth surface and proximal non-cavitated caries lesions in which the porosities of enamel lesion are infiltrated with a low viscosity resin, a technique known as “resin infiltration” creating a diffusion barrier within the lesion without establishing any material on the enamel surface.^1,6^ Thus, resin infiltration can delay the time for restoration placement.

White marks on anterior teeth can be unsightly and patients often seek treatment to have these marks eradicated. White marks associated with the presence of tissue porosity can appear as white patches, white line/stripes, faint white lines, and white speckled lesions (Table 1).

### Etiology of White Marks

There is a wide array of treatments available including whitening as the first choice by Greenwall^7^ and bonding over the mark as the last option, a technique using resin infiltration has been introduced by Munoz et al.^8^ The low viscosity resin infiltrant was used to occlude the pores within the hypomineralised lesion, which acts as diffusion pathways for acids and dissolved minerals, thus sealing these pathways. Thus, the caries infiltration can also be used to camouflage aesthetically disfiguring white spot lesions on buccal surfaces.^9^

**Table 1:** Etiology of white marks

*Type of white mark*	*Etiology*
1. White patches	Trauma to the primary dentition
2. Multiple lesions: brown and white Discolourations	Fluorosis
3. White speckled lesions: mottled enamel	Fever during development
4. Faint white lesions, some black edges	Demineralization lesions after removal of orthodontic brackets
5. Enamel defects and white lesions in deciduous incisors and molars	Ceoliac disease, molar incisor hypoplasia
6. White spot or enamel hypoplasia	Preterm birth

## MATERIALS AND METHODS

The PubMed database research of relevant scientific articles on the effect of resin infiltration on caries lesion progression and esthetics. The search was undertaken with the following keywords: “resin infiltration, dental caries”, “resin infiltration, caries lesions”, “resin infiltration, esthetics,” and “Icon DMG.” The search was limited to articles published in English between the years 2010 and 2015 (Table 2).

## RESULTS

### Resin Infiltration Concept

Resin infiltration technique is a novel technology that bridges the gap between prevention and restoration of carious lesions up to the first third of dentin (D-1) and can camouflage aesthetically disfiguring white lesions on the buccal surface. It is marketed under the name Icon® (DMG America Company, Englewood, NJ) and is described as a micro-invasive technology that fills, reinforces, and stabilizes demineralized enamel without sacrificing the healthy tooth structure.^34,35^

The principle of resin infiltration is to perfuse the porous enamel with resin by capillary action, thereby arresting lesion progression by occluding the microporosities that provide diffusion pathways for the acids and dissolved materials. This technique aims to create a diffusion barrier inside the lesion and not on the lesion surface.^36^ Robinson et al. reported that about 60 ± 10% of the lesion's pore volume had been occupied by resin.^5^ According to Kielbassa et al., resin infiltrates into subsurface lesions and produces resin infiltrated parts of the lesion and the depth of resin infiltration was over 100 μm.^37^

A positive side effect of resin infiltration is that enamel lesions lose their whitish appearance when their microporosities are filled with the resin and look similar to sound enamel. The principle of masking enamel lesions by resin infiltration is based on changes in light scattering within the lesions. Sound enamel has a refractive index (RI) of 1.62. The microporosities of enamel caries lesions are filled with either a watery medium (RI 1.33) or air (RI 1.0). The difference in the refractive indices between the enamel crystals and medium inside the porosities causes light scattering that results in a whitish opaque appearance of these lesions, especially when they are desiccated.^38,39^ The microporosities of infiltrated lesions are filled with resin (RI 1.46) that, in contrast to the watery medium, cannot evaporate. Therefore, the difference in the refractive indices between porosities and enamel is negligible and lesions appear similar to the surrounding sound enamel. As a result, this treatment may be used not only to arrest enamel lesions but also to improve the esthetic appearance of buccal white spots.^38,39^

### Resin Infiltration Technique

Icon^®^ is marketed in two different forms: proximal surface and vestibular surface kits. The usage for both is similar except for the need for separation in the case of proximal lesion treatment. Since the surface layer of enamel caries lesions has a lower pore volume compared to that of the lesion body underneath, it forms a barrier that might hamper the infiltration of resin into the lesion body. Therefore, a preparation phase is required where the surface of the teeth is cleaned and prepared with 15% hydrochloric acid (icon etch) for 2 minutes and stirring the gel from time to time during application with a microbrush. 15% hydrochloric acid gel has been demonstrated to be superior to 37% phosphoric acid gel in removing the mineralized surface layer of natural enamel lesions when applied for 120 seconds. 15% HCL produces a penetration depth of 58 μm, which is more than twice that of phosphoric acid (25 μm), enabling penetration into the deepest part of the lesion, thus eliminating the decalcified areas, preventing further attacks.^40^

Ethanol wet bonding technique is used to desiccate the surface by applying 99% ethanol (Icon Dry) for 30 seconds followed by air drying. It is based on the assumption that it will coax hydrophobic monomers to infiltrate into demineralized wet enamel or dentine, and improve the efficacy of penetration of the hydrophobic infiltrate (TEGDMA) to get a well-defined, resin-infiltrated layer. This technique involves slowly replacing water within the demineralized collagen matrix with ascending concentrations of ethanol, allowing the latter to penetrate the collagen matrix without causing additional shrinkage of the interfibrillar spaces, thus preventing the phase separation of hydrophobic resin monomers.^41,42^

Icon resin, composed of tetraethylene glycol dimethacrylate, is applied on the lesion surface using a microbrush and allowed to penetrate for three minutes. The excess is removed using a cotton roll and light cured. Repeated application for another one minute is performed and then the resin is light cured again. The resin is applied twice because of the shrinkage of the material after the first application, resulting in the generation of space that can be then occluded by a second application. The excess resin is then removed and the surface is polished.^13,43^

The practitioner should select the cases carefully. Resin infiltration technique can treat a smaller white mark much easier than a larger patch. Medium-to-large size patches may require two treatments. If the lesion is very deep, then it is advisable to sandblast the white area prior to applying the hydrochloric acid as an etch to the tooth. The sandblasting helps to open up the enamel tubules so that better penetration of the hydrochloric acid can be achieved.^44^ Teeth with brown discoloration may not be good candidates for resin infiltration, since the later will not mask the brown color and, in fact, it may saturate the color and make it look worse clinically. Microabrasion or conventional resin restorations may be better options for treating teeth with brown discoloration.^39^

### Resin Infiltration in Primary Teeth

The management of non-cavitated caries lesions using the resin infiltration technique in primary teeth differs from that in permanent teeth. Firstly, primary enamel is less mineralized, more porous and aprismatic when compared to permanent enamel. As a result, the diffusion coefficient seems to be greater in primary enamel. Secondly, the proximal surface layer is less mineralized and thinner in primary molars compared to the permanent ones and thus, the rate of progression of proximal caries in primary molars is significantly higher than that in the permanent ones.^44^

**Table 2:** Research studies on resin infiltration therapy

*Reference*	*Type of study*	*Type of teeth*	*Condition*	*Follow-up*	*Outcome*
Ekstrand KR et al. 2010^10^	*In vivo*	Deciduous molars	Hypomineralization	1 year	Resin infiltration in conjunction with fluoride varnish seems promising for controlling proximal lesion progression on deciduous molar teeth
Meyer-Lueckel H et al. 2010^11^	*In vitro*	Permanent posterior teeth	Hypomineralization	–	A solvent-free resin mainly consisting of TEGDMA seems to be the best resin that is capable of penetrating almost completely into enamel parts of natural caries lesions
Belli R et al. 2011^12^	*In vitro*	Bovine incisors	Hypomineralization	–	Infiltration therapy has wear resistance to toothbrush abrasion
Kim S et al. 2011^13^	*In vivo*	Permanent anterior teeth	Developmental defect of enamel and post-orthodontic hypomineralization	1 week	The masking effect is complete in some cases but not in others
Wiegand A et al. 2011^14^	*In vitro*	Bovine incisors	Sound and demineralized enamel	–	The use of resin infiltration before application of a conventional adhesive do not impair bonding to sound and demineralized enamel
Meyer-Lueckel H et al. 2011^15^	*In vitro*	Permanent posterior teeth	Hypomineralization	–	3 min application of an infiltrant seems to be sufficient to achieve an almost complete penetration of enamel caries
Paris S et al. 2011^16^	*In vitro*	Permanent molars and premolars	Hypomineralization	–	Resin infiltrant penetrates most parts of the demineralized enamel but is not capable of filling up cavities
Hammad SM et al. 2012^17^	*In vivo*	Permanent incisors	Post-orthodontic hypomineralization	–	Aesthetic improvement can be achieved with resin infiltration therapy
Meyer-Lueckel H et al. 2012^18^	*In vivo*	Permanent posterior teeth	Hypomineralization	3 years	Resin infiltration is an efficacious method to hamper progression of non-cavitated proximal lesions extending radiographically up to the outer third of dentin
Martignon S et al. 2012^19^	*In vivo*	Permanent posterior teeth	Hypomineralization	3 years	Infiltration and sealing are significantly better than placebo treatment for controlling caries progression on proximal lesions
Nadia Malek Taher 2012^20^	*In vitro*	Premolars	Sound teeth	–	Microhardness of the enamel surface treated with Icon was approximately the same as that of sound enamel and showed a clinically acceptable surface roughness
Paris S et al. 2013^21^	*In vitro*	Bovine teeth	Hypomineralization	–	Polished infiltrated lesions are more resistant to staining
Jia L et al. 2013^22^	*In vitro*	Bovine incisors	Sound teeth	–	Dentin contamination with the resin infiltrant system impair the shear bond strength of conventional dental adhesives
Paris S et al. 2013^23^	*In vitro*	Bovine incisors	Hypomineralization	–	Resin infiltration significantly improves microhardness and demineralization resistance of enamel lesions; these effects are significantly enhanced if resin is applied twice
Araujo GS et al. 2013^24^	*In vitro*	Third molars	Hypomineralization	–	Solvents added to monomer blends result in decreased properties of the resin. The addition of hydrophobic monomers and solvents into TEGDMA blends does not improve the penetration depth of the infiltrants
Paris S et al. 2013^25^	*In vitro*	Permanent molars and premolars	Hypomineralization	–	Application of either ethanol or acetone, followed by air-drying, is suitable to prepare caries lesions for resin infiltration
Knosel et al. 2013^26^	*In vivo*	Permanent anterior teeth	Post-orthodontic hypomineralization	6 months	Resin infiltration improves the esthetic appearance of demineralized teeth
Naidu et al. 2013^27^	*In vitro*	Bovine teeth	Sound and hypomineralized enamel	–	Preconditioning with infiltrant system increase the shear bond strength of most orthodontic resin cements while decreasing the risk of enamel fracture at debonding
Tirlet G et al. 2013^27^	*In vivo*	Permanent anterior teeth	Fluorosis and traumatic hypomineralization	19 months	Resin infiltration could be a promising minimally invasive treatment in fluorosis and traumatic hypomineralization
Munoz et al. 2013^28^	*In vivo*	Permanent anterior teeth	Fluorosis and traumatic hypomineralization	4 months	Resin infiltration can be considered a minimally invasive procedure for mild-to-moderate fluorosis and hypoplasia stains related to traumatic dental injuries
Liu Yonghong et al. 2013^29^	*In vitro*	primary molars and permanent posterior teeth	Proximal lesions	–	Better penetration abilities of resin infiltration in primary molars are shown in lesions confined to the outer half of enamel than those in permanent posterior teeth.
Wolfgang H Arnold et al., 2014^30^	*In vitro*	Permanent posterior teeth	Hypomineralization	–	Artificial lesions were completely penetrated by the resin and that artificial caries-like lesions can be used, within the limits of the shallow artificial lesions, to perform experimental studies on resin infiltration into lesions
M. B. Altarabulsi et al. 2014^31^	*In vivo*	Deciduous and permanent teeth	Proximal lesion in enamel or in the outer third of dentin	12 months	Caries infiltration hampers the progression of initial proximal lesion extending radiographically in the enamel or the outer third of dentin
Priya Subramaniam et al. 2014^32^	*In vitro*	Sound premolars	Hypomineralization	–	The maximum depth of penetration of the resin material was 6.06 ± 3.32 μm
Monica Almeida Tostes et al. 2014^33^	*In vitro*	Bovine teeth	Hypomineralization	–	The untreated lesion showed lower hardness values using a nanoindenter equipment for distances near the outer surface of enamel
Soley Arslan et al. 2015^34^	*In vitro*	Sound permanent incisors	Hypomineralization	–	Resin infiltration technique showed an increase in microhardness and a decrease in roughness of demineralized enamel surfaces, coupled with low bacterial adhesion and thus capable of arresting initial enamel carious lesions

In an *in vitro* study by Paris S et al., primary teeth exhibited better infiltrant penetration than permanent teeth, after 1 minute application of resin.^45^ On the other hand, 3–5 minutes are required to almost completely infiltrate a natural lesion in permanent teeth with a lesion extended to the inner half of enamel, whereas, one-minute application resulted in only superficial infiltration.^14^

Following 5 minute resin application, Liu et al. found no significant differences in the overall penetration between primary and permanent molar lesions but the penetration abilities of primary molars were slightly higher than those of permanent teeth in lesions confined to the outer half of enamel.^29^ Ekstrand et al. conducted a split-mouth study for one year to assess the efficacy of resin infiltrated lesions covered by fluoride varnish vs fluoride varnish treatment only on the proximal lesions of deciduous molars. Lesion progression was assessed clinically and radiographically. Proximal caries in primary molars treated by resin infiltration and fluoride varnish progressed significantly lesser (23%) than those treated with fluoride varnish only (61%) after one year.^10^

### Advantages of Resin Infiltration

Resin infiltration has made possible an innovative way of treating initial carious lesions that fits perfectly with the concept of minimal intervention dentistry. Infiltration of carious lesions represents a new approach to the treatment of non-cavitated lesions of proximal and smooth surfaces of deciduous and permanent teeth up to the first third of dentin (D-1 level). From the foregoing review, it seems clear that the resin infiltration technique bears several advantages as follows:

- Noninvasive treatment, preserving tooth structure;
- Achieved in a single visit;
- Mechanical stabilization of demineralized enamel;
- Deeper penetration into porous demineralized areas;
- Arrest/retardation of lesion progress;
- Minimized risk of secondary caries;
- No risk of postoperative sensitivity and pulpal inflammation;
- Reduced risk of gingivitis and periodontitis;
- Rmproved esthetic outcome when used as a “masking” resin on demineralized labial surfaces (white spot lesions, i.e. with orthodontic patients);
- High patient acceptance.

While this therapy can rightly be categorized as minimum intervention dentistry, clinical experience is limited and further controlled clinical trials are required to assess its long-term results.^46^

### Aesthetic Outcome of Resin Infiltration Therapy

Cosmetics and esthetics are current trends of dental industry. As more and more patients are demanding for minimally invasive cosmetic enhancement without anesthesia and drilling, the technique of resin infiltration may be considered as a microinvasive treatment of smooth-surface white spot lesions and also one that allows for the recovery of natural tooth appearance.

The porosity created by the initial demineralization of a caries process changes the refractive index of enamel, resulting in a white coloration in the incipient lesion. The resin infiltration technique has an additional positive effect on esthetics in which the penetration and polymerization of the low viscous resin inside the lesion body allows a change of the lesion's whitish appearance to the natural enamel appearance.^18,47^ Knosel et al. in a clinical trial with patients with white spot lesions treated after the removal of braces reported that there were no statistically significant differences in the color of the infiltrated resin during a 6 month follow-up, confirming the aesthetic effect of this treatment.^26^ However, Kim S et al. in his clinical study on assessing the effectiveness of masking white spot enamel lesions using resin infiltration found that among the 20 teeth with the developmental defect of enamel, 5 teeth (25%) were classified as completely masked, whereas 7 (35%) and 8 teeth (40%) were partially masked and unchanged, respectively. Among the 18 teeth with decalcification, 11 teeth (61%) were completely masked, 6 teeth (33%) were partially masked, and 1 tooth (6%) was unchanged. In some teeth, the result was more improved after 1 week than immediately after infiltration.^13^ Since just a few articles mention more than the immediate aesthetic outcome, there is an evident need for more clinical studies demonstrating long-term aesthetic results of resin infiltration therapy.

### Limitations of Resin Infiltration

Even though the resin infiltration technique has opened up a new range of options for minimal invasive treatment of white spots, there is the need to mention few reasons that may affect the success of the treatment.

- Inefficient isolation;
- Incomplete resin polymerization;
- Depth of the lesion.^20,48,49^

ICON works on the principle of infiltration and requires a very dry field. Apart from keeping the environment moisture-free, additional steps must be taken to dry the lesion. This is accomplished by treating the lesion area with alcohol, which evaporates the water within the porosities, which can inhibit the process of infiltration.

The greater the depth of the carious lesion, the lower will be the probability of achieving a complete infiltration. Extensive lesions are also associated with a higher polymerization shrinkage and the consequent appearance of porosities and cracks.^22^ The infiltration of cavitated lesions does not produce satisfactory results, taking into account the weak capillary action of the resin into these lesions.^15^

Ekstrand et al. evaluated the effectiveness of the treatment of proximal lesions of temporary molars with resin infiltration. The reported rate of failure after one year (23 vs 62% in the control group) was higher than that reported in other studies after the same period of follow up. However, unlike those, the sample used by Ekstrand et al. composed of only children with moderate to high risk, which may partially explain the results.^10^

## CONCLUSION

Caries resin infiltration represents a new concept in dentistry and therefore needs to be better investigated. Based on the available laboratory and clinical studies, it seems convincing that the resin infiltration of enamel lesions should reduce (or even stop) the progress of white spot lesions. This technique is considered to be microinvasive and might bridge the gap between non-invasive and minimally invasive treatment of initial dental caries, postponing, as long as possible, the need for restoration.
